# Buffalo Hospital of the Sisters of Charity

**Published:** 1852-01

**Authors:** 


*Buffalo Hospital of the Sisters of Charity.*—Our last number contained an annual statistical report of the cases treated at this noble charity. About eight hundred patients have found an asylum at the institution during the past year. At the present moment it contains about one hundred and twenty inmates. When it is recollected that, prior to the establishment of this hospital, a little more than three years since, by the venerable Catholic Bishop of the diocese of Buffalo, (the Rt. Rev. John Timon,) aided by the Sisters of Charity, no place of retreat for the sick poor of this city existed, save the almshouse, some conception may be formed of the service it has rendered in behalf of Christian philanthropy. What would have been the fate of the thousands who have already experienced the benefits of the institution during the brief period of its existence, had there been no such charity? Destitution and neglect, or the degradation of pauperism would have been, as before, the alternatives. While many have been spared for the duties and enjoyments of life and health, still more have been saved from that loss of character and self-respect which almost necessarily belongs to the stigma of having been forced to resort to the *poor-house.* In this latter aspect, the charity of hospitals is especially manifest. Let the accommodations and comforts of a pauper institution be ever so great, that charity is equivocal which so degrades those whose chief misfortunes are poverty and disease. Hospitals impose no degradation. Herein consists their strongest claim on the encouragement and support of the benevolent.

As regards the alms house in this county, we take great pleasure in bearing testimony to the improvements which have been effected, and the diminished rate of mortality, under the auspices of the present excellent keeper and physician, Dr. J. B. Pride. Still, for reasons just intimated, like all such establishments, it should be restricted, as far as possible, to those who have nothing to lose by becoming paupers. Good policy, not less than humanity, dictates this course.

Subjects pertaining to hospitals and pauper relief are not only appropriate, but commend themselves especially to the attention of medical men. We have heretofore submitted some views on these subjects, and we purpose, ere long, to renew the discussion of certain topics in which, as it seems to us, physicians should feel an interest. Want of time and space compels us to forego, for the present, this intention.

As a field for clinical instruction, the Buffalo Hospital of the Sisters of Charity is, perhaps, not excelled by any institution in the country. The number and variety of diseases are abundantly adequate, and the number of cases is not so great as to embarrass or overwhelm the efforts of teacher and pupil. It is conceded, and it is obvious, that for purposes of scientific observation and teaching, a moderately large hospital presents advantages which do not belong to a mammoth establishment embracing more cases than can be satisfactorily studied and treated. Moreover, in the wards of an overcrowded institution, the absence of favorable hygienic circumstances is liable to affect the progress and character of many diseases, materially impairing their value as exemplifying the natural history of these diseases, and illustrating the operation of therapeutic agencies. A medical experience based on facts thus acquired, would prove a fallacious guide in the practice of medicine.

The Buffalo Hospital, having no permanent endowment, depends, for its revenues, on various sources, which are somewhat uncertain. A small compensation is derived from a portion of the patients received. The emigrant fund supplies a small stipend. The students of the Medical College who visit the wards, pay a moderate sum. Some county poor are received at less than a dollar a week, and the remainder is made up of the donations of those benevolent persons who avail themselves of the privilege here offered of contributing to a charitable object, which, of all others, is least open to distrust. It would be impossible to meet the pecuniary wants of the hospital from these several sources, were it not that, aside from the utmost prudence consistent with the fullest justice to the sick, the expenses are comparatively small, owing to the gratuitous services of the Sisters of Charity, who, for devoting their lives to the care of the sick, only claim in return the simplest necessaries of life. What a contrast to that philanthropy which expends its energies in loud professions and empty declarations, is afforded by the quiet, unobtrusive, practical, and life-devoted efforts of these angels of mercy, who, in looking for their reward hereafter, receive the approbation of conscience, and thereby the highest enjoyment which belongs to this world!

